# From Rheumatology 1.0 to Rheumatology 4.0 and beyond: the contributions of Big Data to the field of rheumatology

**DOI:** 10.31138/mjr.30.1.3

**Published:** 2019-03-28

**Authors:** Nicola Luigi Bragazzi, Giovanni Damiani, Mariano Martini

**Affiliations:** 1 Postgraduate School of Public Health, Department of Health Sciences (DISSAL), University of Genoa, Genoa, Italy,; 2 Clinical Dermatology, IRCCS Istituto Ortopedico Galeazzi, Department of Biomedical, Surgical and Dental Sciences, University of Milan, Milan, Italy,; 3 Young Dermatologists Italian Network (YDIN), GISED, Bergamo, Italy,; 4 Department of Dermatology, Case Western Reserve University, Cleveland, OH, USA,; 5 Department of Health Sciences, Section of Medical History and Ethics, University of Genoa, Italy,; 6 UNESCO CHAIR Anthropology of Health - Biosphere and Healing System, University of Genoa, Italy

In their paper, Mansour and colleagues^1^ have explored the relationship between rheumatoid arthritis (RA) and venous thromboembolism (VTE), an association which has been relatively overlooked by the existing scholarly literature, despite mounting evidence suggesting a link between coagulation factors and pro-inflammatory molecules. The majority of the previous studies has employed small sample sizes, being generally statistically underpowered, except for few notable exceptions. Utilizing a large nation-wide database, comprising of 11,782 patients with RA, and 57,973 age- and gender-matched controls, Mansour and co-workers were able to perform a scientifically sound and robust study, which could capture a significant association between RA and VTE. Among RA patients, VTE episodes had a rate of 6.92% versus 3.18% among the controls (statistically significant with a p-value <0.001). At the multivariate logistic regression, an odds-ratio (OR) of 2.23 (95% confidence interval or CI 2.05–2.43) and an OR of 1.60 (95%CI 1.44–1.78), both statistically significant, were computed in the model not adjusted for C-reactive protein (CRP) and in the model corrected for CRP, respectively.

In a previously published systematic review of the literature and meta-analysis,^2^ a pooled risk ratio of 1.90 (95%CI 1.76–2.06) of VTE episodes in RA patients had been found. Mansour et al.^1^ have replicated this finding, confirming the existence of such link. Taken together, all these results warrant the role of thromboprophylaxis in immune mediated disorders, such as RA. Despite the existence of tools like the “Padua Prediction Score”, there is an urgent need of new validated instruments and scores which assess the risk of VTE episodes in hospitalized patients, giving more weight to current inflammatory rheumatologic conditions, and, therefore, potentially estimating the risk of VTE in a more realistic fashion.

In their papers, Mansour and colleagues^1^ have used an approach different from the classical one: instead of utilizing a hypothesis-driven conceptual framework, they have relied upon data-driven techniques, including massive data mining. Real-world experiences (RWEs) making use of real-world data (RWD) of patients treated in real-world settings (RWS) are emerging as a vital, integral component of the process of healthcare decision-making, shaping and informing new real-world evidence (RWE).^3,4^ The use of large clinical databases and registries is paving the way for a new stratified and precision medicine,^5^ in which data-driven disease phenotyping and profiling play a major role.^6–8^ This represents an authentic paradigm shift with regards to the classical “one-size-fits-it-all” framework, favoring the rise of a personalized rheumatology, in which diagnosis and treatment are tailored to the specific features and (biological, genetic, epigenetic, and behavioral) make-up of the patient.

Rheumatology has a long, millennial, rich tradition. Its history dates back to the Indian Ayurvedic physician Charaka (approximately 300–200 b.c.), who was an authentic pioneer in the field of rheumatology. In the treatise “*Charaka Samhita*”, he described and characterized different clinical phenotypes of arthritis and RA (*vishkantha* in Sanskrit), whereas Hippocrates (450–380 b.c.) contributed to the diagnosis of gout and probably described also episodes of rheumatic fever. Galen (129–216 a.d.) coined the term “rheumatismus”.^9–11^

These descriptions and reports characterized the “prehistory” of rheumatology (a period that we term as “rheumatology 1.0”), which was further advanced by the work of the prominent Flemish physician William Heberden (1710–1801), who is considered the father of the classical rheumatology (the so-called “rheumatology 2.0”). He was, indeed, the first physician to clinically distinguish between osteoarthritis (OA) and gout. In his book entitled “Commentaries on the history and cure of disease” he reported the “*digitorum nodi*”, which were named after him, being currently known as Heberden’s nodes, and being pathognomonic of OA.^12^

The work of Heberden was preceded by the work of the French physician Guillaume de Baillou (also known as *Ballonius*; 1538–1616)^13^ and the English physician Thomas Sydenham (1624–1689).^14^ de Baillou, besides describing the first epidemic of pertussis, characterized the rheumatic fever. Sydenham described several rheumatologic disorders, including RA, gout, rheumatic fever, scorbutical rheumatism and *chorea minor*, which he proposed to term as Saint Vitus’ dance. Other prominent rheumatologists were the French doctor Augustin Jacob Landré-Beauvais (1772–1840), who was only a 28-year-old medical resident when, at the beginning of the XIX^th^ century, he described a clinical case of RA, which is the first officially accepted medical record of RA.^15^ Alfred Garrod (1819–1907), discoverer of abnormal levels of uric acid in the blood of gout patients, and Archibald Garrod (1857–1936), co-author of the “Treatise on Rheumatism and Rheumatoid Arthritis”, further advanced the field of rheumatology, contributing to the clinical diagnosis of RA, better specifying its diagnostic criteria. John Kent Spender introduced the word “osteoarthritis” in 1886,^16^ or, according to some scholars, re-introduced and popularized a term coined by the German surgeon Richard von Volkmann (1830–1889).^17^

The XIX^th^ century has been a fervent period, rich in discoveries and scientific achievements. For instance, Dundas described rheumatic fever,^18^ Money the myocardial granulomas in 1883, the German physician Aschoff (1866–1942) the nodules named after him (Aschoff’s nodules), whereas the association between chorea and rheumatic fever was reported independently by the physicians Bright and See (in 1831 and in 1850, respectively).^9^ The American physician Homer Fordyce Swift (1881–1953), besides describing syphilis and discovering a treatment for cerebrospinal syphilis (which was named after him and Arthur Ellis) wrote extensively on the rheumatic fever and streptococcal infections. In 1928, he was able to link rheumatic fever with *Streptococcus*,^19^ which was later identified as causative agent by the English physician Collis and the American Coburn in 1931.^9^ One year after, in 1932, Todd introduced the anti-streptolysin test.^20^

In 1872, the Hungarian physician and dermatologist Moritz Kaposi (1837–1902) described the systemic nature of systemic lupus erythematosus, which was later confirmed by the Canadian physician Osler (1849–1919) in 1900.^9^ In 1909 Nichols and Richardson were able to differentiate OA and RA from a clinical standpoint, distinguishing between the degenerative and proliferative phenotypes of arthritis.^9^ Some years earlier, in 1884, the French physician Bouchard (1837–1915) had described the bony outgrowths at proximal inter-phalangeal joints (named after him, Bouchard’s nodes), which represent a pathognomonic sign of OA.

The classic Reiter’s triad (comprising of reactive arthritis, conjunctivitis and urethritis), occurring after uro-genital infections or dysenteric episodes,^21^ has been described during the First World War by the German physician Hans Reiter (1881–1969) and, nearly contemporarily and independently, by the French Nöel Fiessinger (1881–1946) and Edgar Leroy.^22^

Initially borne within internal medicine, rheumatology was recognized as a medical specialty *per se* with its own dignity (“rheumatology 3.0”), by Bernard Comroe and Joseph Lee Hollander (1910–2000), who coined the term “rheumatologist” in 1940 and co-authored the textbook “Arthritis and Allied Conditions”. Furthermore, Comroe opened the first arthritis clinics in the USA.^9,23^

This era is characterized by new diagnostic criteria and nosological definitions and classifications. For instance, in 1942, the concept of “connective tissue disorders” was introduced and developed by Paul Klemperer (1887–1964). In 1963, the term “ankylosing spondylitis” was adopted, together with the Rome’s criteria.

At the turn of the XX^th^ century, biological advancements and technological achievements have further revolutionized rheumatology, which could now enter the fourth phase (“rheumatology 4.0”),^24^ characterized by new sophisticated techniques of imaging, functional genomics and post-genomics specialties (proteomics, metabonomics, microbiomics, among the others), the advent of electronic medical records (EMRs) and clinical registries, evidence-based medicine (EBM), with large epidemiological surveys and multi-center studies, including randomized controlled trials (RCTs), as well as wearable sensors and novel data streams (NDS, such as social networks and the internet-based data). Altogether this has provided scholars with an unprecedented wealth of data (the so-called “Big Data”).^25–33^

Big Data, classically characterized by 3 Vs (volume, velocity and variety), are massive datasets whose size and volume are so large that they exceed the computational capacity of conventional relational database systems to capture, store, manage and analyze them. In the medical field, Big Data can be of different types, depending on the source that generates them: they can be of molecular type (the so-called omics data, generated by high-throughput molecular assays of the latest generation), administrative (socio-economic and demographic data, the so-called transaction data), of instrumental/laboratory type (M2M, known as machine-to-machine data), of clinical type (EMRs, and other clinical application data) and social/behavioral (what the patient knows about the disease and what they search for online, the so-called web and social media-generated data).

In the field of healthcare, Big Data can be used for different purposes: including clinical epidemiology, risk prediction, diagnostic and prognostic accuracy, improvement of the clinical outcomes, assessment of appropriateness of pharmacological prescriptions, and implementation and monitoring of the quality of the diagnostic-therapeutic care, among others.

Specifically, in the field of rheumatology, Mansour and colleagues^1^ have contributed to write a new page of Rheumatology 4.0. In conclusion, Big Data-based rheumatology (“rheumatology 4.0”) appears to be a promising approach to rheumatologic diseases, even if the revolution has only just begun!

## References

[1] Mansour R Azrielant S Watad A Tiosano S Yavne Y Comanesther D Venous thromboembolism events among RA patients. Mediterr J Rheumatol 2019;30(1):38–43. 10.31138/mjr.30.1.38 PMC7045915 32185341

[2] Ungprasert P Srivali N Spanuchart I Thongprayoon C Knight EL Risk of venous thromboembolism in patients with rheumatoid arthritis: a systematic review and meta-analysis. Clin Rheumatol 2014 3;33(3):297–304. [10.1007/s10067-014-2492-7] [PMID: ] 24424839

[3] Misra DP Agarwal V Real-world evidence in rheumatic diseases: relevance and lessons learnt. Rheumatol Int 2019 3;39(3):403–416. [10.1007/s00296-019-04248-1] [PMID: ] 30725156

[4] Miksad RA Abernethy AP Harnessing the Power of Real-World Evidence (RWE): A Checklist to Ensure Regulatory-Grade Data Quality. Clin Pharmacol Ther 2018 2;103(2):202–5. [10.1002/cpt.946] [PMID: ] [PMCID: ] 29214638 PMC5814721

[5] Cho SK Sung YK A paradigm shift in studies based on rheumatoid arthritis clinical registries. Korean J Intern Med 2019 2 18. [10.3904/kjim.2018.440] [PMID: ] 30759964 PMC6718765

[6] Brito-Zerón P Acar-Denizli N Ng WF Zeher M Rasmussen A Mandl T How immunological profile drives clinical phenotype of primary Sjögren’s syndrome at diagnosis: analysis of 10,500 patients (Sjögren Big Data Project). Clin Exp Rheumatol 2018 May-Jun;36 Suppl 112 (3):102–12. [PMID: ] 30156539

[7] Schmajuk G Yazdany J Leveraging the electronic health record to improve quality and safety in rheumatology. Rheumatol Int 2017 10;37(10):1603–10. [10.1007/s00296-017-3804-4] [PMID: ] [PMCID: ] 28852846 PMC5693630

[8] Cook JA Collins GS The rise of big clinical databases. Br J Surg 2015 1;102(2):e93–e101. [10.1002/bjs.9723] [PMID: ] 25627139

[9] Deshpande S History of rheumatology. Med J DY Patil Univ 2014;7:119–23.

[10] Entezami P Fox DA Clapham PJ Chung KC Historical perspective on the etiology of rheumatoid arthritis. Hand Clin 2011 2;27(1):1–10. [10.1016/j.hcl.2010.09.006] [PMID: ] [PMCID: ] 21176794 PMC3119866

[11] Sakkas LI Tronzas P The Greek (Hellenic) rheumatology over the years: from ancient to modern times. Rheumatol Int 2019, in press. [10.1007/s00296-019-04261-4] 30805680

[12] Joshi VR Poojary VB William Heberden (elder) (1710–1801). J Assoc Physicians India 2013 12;61(12):946–7. [PMID: ] 24968564

[13] Baruch JZ [Guillaume de Baillou, precursor of modern rheumatology. Some biographical data with contemplations]. Ned Tijdschr Geneeskd 1965 10 30;109(44):2086–91. [PMID: ] 5321429

[14] Stochik AM Zatravkin SN [The contribution of T. Sydenham (1624–1689) to the formation of modern Western medicine (on the occasion of the 390th anniversary of his birth)]. Ter Arkh 2014;86(8):138–42. [PMID: ] 25306762

[15] Landré-Beauvais AJ The first description of rheumatoid arthritis. Unabridged text of the doctoral dissertation presented in 1800. Joint Bone Spine 2001 3;68(2):130–43. [PMID: ] 11324929 10.1016/s1297-319x(00)00247-5

[16] Spender JK On some Hitherto Undescribed Symptoms in the Early History of Osteoarthritis. The So-Called Rheumatoid Arthritis. Br Med J 1888 4 14;1(1424):781–3. [PMID: ] [PMCID: ] 20752255 10.1136/bmj.1.1424.781 PMC2197518

[17] Dobson GP Letson HL Grant A McEwen P Hazratwala K Wilkinson M Defining the osteoarthritis patient: back to the future. Osteoarthritis Cartilage 2018 8;26(8):1003–7. [10.1016/j.joca.2018.04.018] [PMID: ] 29775734

[18] Dundas D An account of a peculiar Disease of the Heart. Med Chir Trans 1809;1:37–46. [PMID: ] [PMCID: ] 20895121 10.1177/095952870900100105 PMC2128801

[19] Dochez AR Homer Fordyce Swift, 1881–1953. Trans Assoc Am Physicians 1954;67:25–7. [PMID: ] 13216807

[20] Lawy HS Use and interpretation of the antistreptolysin test. Ann Rheum Dis. 1960;19:42–7. [PMID: ] [PMCID: ] 14414765 10.1136/ard.19.1.42 PMC1007358

[21] Wu IB Schwartz RA Reiter’s syndrome: the classic triad and more. J Am Acad Dermatol 2008 7;59(1):113–21. [10.1016/j.jaad.2008.02.047] [PMID: ] 18436339

[22] Pasero G Marson P. [Wars in the history of rheumatology]. Reumatismo 2007 Oct-Dec;59(4):332–7. [PMID: ] 18157291 10.4081/reumatismo.2007.332

[23] Benedek TG History of Rheumatic Diseases. In: (Klippel J.H., Ed.) “Primer on the Rheumatic diseases”. Atlanta, Arthritis Foundation,1997;11:1–5.

[24] Burmester GR Rheumatology 4.0: big data, wearables and diagnosis by computer. Ann Rheum Dis 2018 7;77(7):963–5. [10.1136/annrheumdis-2017-212888] [PMID: ] [PMCID: ] 29802224 PMC6029631

[25] Burmester GR Häupl T Big data-also relevant in rheumatology? Z Rheumatol. 2018 4;77(3):192–4. 29619762 10.1007/s00393-018-0437-2

[26] Sfikakis PP Bournia VK Sidiropoulos P Boumpas DT Drosos AA Kitas GD Biologic treatment for rheumatic disease: real-world big data analysis from the Greek country-wide prescription database. Clin Exp Rheumatol 2017 Jul-Aug;35(4):579–85. [PMID: ] 28281458

[27] Brito-Zerón P Acar-Denizli N Zeher M Rasmussen A Seror R Theander E Influence of geolocation and ethnicity on the phenotypic expression of primary Sjögren’s syndrome at diagnosis in 8310 patients: a cross-sectional study from the Big Data Sjögren Project Consortium. Ann Rheum Dis 2017 6;76(6):1042–50. [10.1136/annrheumdis-2016-209952] [PMID: ] 27899373

[28] Symmons DP Epidemiology research in rheumatology-progress and pitfalls. Nat Rev Rheumatol 2015 11;11(11):631–8. [10.1038/nrrheum.2015.92] [PMID: ] 26150125

[29] Gonzalez A Valdes AM Big data boost for osteoarthritis genetics. Nat Rev Rheumatol 2018 7;14(7):387–88. [10.1038/s41584-018-0023-7] [PMID: ] 29899548

[30] Landewé RBM van der Heijde D “Big Data” in Rheumatology: Intelligent Data Modeling Improves the Quality of Imaging Data. Rheum Dis Clin North Am 2018 5;44(2):307–15. [10.1016/j.rdc.2018.01.007] [PMID: ] 29622297

[31] Winter DR Thinking BIG rheumatology: how to make functional genomics data work for you. Arthritis Res Ther 2018 2 12;20(1):29. [10.1186/s13075-017-1504-9] [PMID: ] [PMCID: ] 29433549 PMC5810031

[32] Ramos-Casals M Brito-Zerón P Kostov B Sisó-Almirall A Bosch X Buss D Google-driven search for big data in autoimmune geoepidemiology: analysis of 394,827 patients with systemic autoimmune diseases. Autoimmun Rev 2015 8;14(8):670–9. [10.1016/j.autrev.2015.03.008] [PMID: ] 25842074

[33] Bragazzi NL Watad A Brigo F Adawi M Amital H Shoenfeld Y Public health awareness of autoimmune diseases after the death of a celebrity. Clin Rheumatol 2017 8;36(8):1911–7. [10.1007/s10067-016-3513-5] [PMID: ] 28000011

